# Access to artesunate-amodiaquine, quinine and other anti-malarials: policy and markets in Burundi

**DOI:** 10.1186/1475-2875-10-34

**Published:** 2011-02-10

**Authors:** John H Amuasi, Graciela Diap, Samuel Blay-Nguah, Isaac Boakye, Patrick E Karikari, Baza Dismas, Jeanne Karenzo, Lievin Nsabiyumva, Karly S Louie, Jean-René Kiechel

**Affiliations:** 1 Komfo Anokye Teaching Hospital (KATH), Kumasi, P.O. BOX KS 1934, Ghana; 2 Drugs for Neglected Diseases initiative (DNDi), Geneva, 1202, Switzerland; 3 University of Minnesota School of Public Health, PhD Program, Division of Health Policy and Management, Mayo Memorial Building, 420 Delaware Street S.E., Minneapolis, MN 55455, USA; 4 National Malaria Control Programme, Ministry of Health, Bujumbura, Burundi; 5 Department of Clinical Research, Faculty of Infectious & Tropical Diseases, London School of Hygiene and Tropical Medicine, London, UK

## Abstract

**Background:**

Malaria is the leading cause of morbidity and mortality in post-conflict Burundi. To counter the increasing challenge of anti-malarial drug resistance and improve highly effective treatment Burundi adopted artesunate-amodiaquine (AS-AQ) as first-line treatment for uncomplicated *Plasmodium falciparum *malaria and oral quinine as second-line treatment in its national treatment policy in 2003. Uptake of this policy in the public, private and non-governmental (NGO) retail market sectors of Burundi is relatively unknown. This study was conducted to evaluate access to national policy recommended anti-malarials.

**Methods:**

Adapting a standardized methodology developed by Health Action International/World Health Organization (HAI/WHO), a cross-sectional survey of 70 (24 public, 36 private, and 10 NGO) medicine outlets was conducted in three regions of Burundi, representing different levels of transmission of malaria. The availability on day of the survey, the median prices, and affordability (in terms of number of days' wages to purchase treatment) of AS-AQ, quinine and other anti-malarials were calculated.

**Results:**

Anti-malarials were stocked in all outlets surveyed. AS-AQ was available in 87.5%, 33.3%, and 90% of public, private, and NGO retail outlets, respectively. Quinine was the most common anti-malarial found in all outlet types. Non-policy recommended anti-malarials were mainly found in the private outlets (38.9%) compared to public (4.2%) and NGO (0%) outlets. The median price of a course of AS-AQ was US$0.16 (200 Burundi Francs, FBu) for the public and NGO markets, and 3.5-fold higher in the private sector (US$0.56 or 700 FBu). Quinine tablets were similarly priced in the public (US$1.53 or 1,892.50 FBu), private and NGO sectors (both US$1.61 or 2,000 FBu). Non-policy anti-malarials were priced 50-fold higher than the price of AS-AQ in the public sector. A course of AS-AQ was affordable at 0.4 of a day's wage in the public and NGO sectors, whereas, it was equivalent to 1.5 days worth of wages in the private sector.

**Conclusions:**

AS-AQ was widely available and affordable in the public and NGO markets of hard-to-reach post-conflict communities in Burundi. However greater accessibility and affordability of policy recommended anti-malarials in the private market sector is needed to improve country-wide policy uptake.

## Background

The East African country of Burundi is one of the smallest countries on the continent, with a population of about 8 million in 2008. Burundi's health infrastructure collapsed during the 13-year civil war that ended in 2005. Presently in post-conflict transition, the country is focussing its efforts in rebuilding its health system and reducing the public health burden of diseases. Malaria is the main cause of morbidity and mortality in Burundi, with an estimated 2.3 million cases and 7,700 deaths in 2006, of which 70% of these deaths were among children less than five years of age [1]. In 2003, Burundi adopted WHO-recommended artemisinin combination therapy (ACT), specifically artesunate-amodiaquine (AS-AQ), as effective first-line treatment for uncomplicated *Plasmodium falciparum *malaria and oral quinine as second-line in the event of treatment failure. This policy was adopted following the 2000/2001 malaria epidemic that was heightened by high parasite resistance to chloroquine [2] and sulphadoxine-pyrimethamine.

Although a new national treatment policy has been adopted, accessibility as defined by availability, pricing, and affordability, to these recommended anti-malarial treatments remains a challenge. A number of studies have evaluated the implementation of ACT policy and access to ACT in the public sector of Burundi soon after policy adoption. After the first nine months of policy implementation, one community-based study found AS-AQ was the most common treatment for children <5 years of age with uncomplicated malaria attending public sector facilities, but coverage was low and the prices patients were paying were ten times higher than the subsidized price [3]. In another report, WHO found no recorded shortage of AS-AQ at the national level in the first year of ACT policy implementation, but 60% of public sector health facilities experienced stock-outs [4]. In addition, 20% of malaria cases were treated with oral quinine as first-line treatment rather than AS-AQ since sales of quinine provided more income to support the provision of services at these facilities under a government cost recovery policy. A nationally representative household survey showed that only 30% of under-fives who were ill with fever in the preceding two weeks received any anti-malarial treatment drugs in Burundi [5]; however, it was not stated whether these were ACT or not.

Specifically, high anti-malarial pricing is an important contributing factor to the lack of access to ACT [6]. In the public and NGO retail sectors, each treatment of AS-AQ should be available at the government subsidized price of 200 Burundian Francs (FBu - the equivalent of US$0.16). In addition, treatment should be free for children ≤ 5 years of age. However, it is unknown whether AS-AQ is truly available at these subsidized prices. Although AS-AQ should be readily available in the public sector, it is often more costly in the private sector. Many patients in Africa use the private sector as their primary source of medicines with 50% of febrile episodes reported to be treated in the private sector [7], where marked-up manufacturer's selling prices and final patient range from 56 to 358%, making treatment unaffordable [8]. On average, households in many malaria endemic countries spend up to 90% of their household expenditure on medicines [7].

The government of Burundi recently procured new fixed-dose ASAQ combination tablets to treat uncomplicated malaria in order to improve prescribing practices, patient compliance and reduce the risk of parasite resistance to AS-AQ. This fixed-dose formulation allows AS and AQ to be taken together in correct proportions in a simplified three-day dose regimen where the most vulnerable population, children under aged five, take only one tablet per day. Since there are no subsidies in the private sector, the government is also considering to market fixed-dose combination ASAQ in the private sector with prices comparable to those in the public sector in a bid to increase nationwide affordability (personal communication with the NMCP of Burundi, 16^th ^February 2009). Without heavy ACT subsidies in both the public and private sectors and other interventions, coverage of effective treatment may not be realized and will remain low [9].

Access to AS-AQ at the local level in Burundi is unknown and it is important that the recently procured fixed-dose ASAQ is effectively distributed to those with the potential to suffer from uncomplicated *P. falciparum *malaria.

The objective of this study was to evaluate access to national policy anti-malarials, AS-AQ and quinine as defined by availability, pricing and affordability in the public, private and NGO retail markets in Burundi. Results from this study will help inform advocacy campaigns and policy recommendations to improve country-wide access to new fixed-dose ASAQ to treat uncomplicated malaria.

## Methods

### Study area

**Figure 1:**
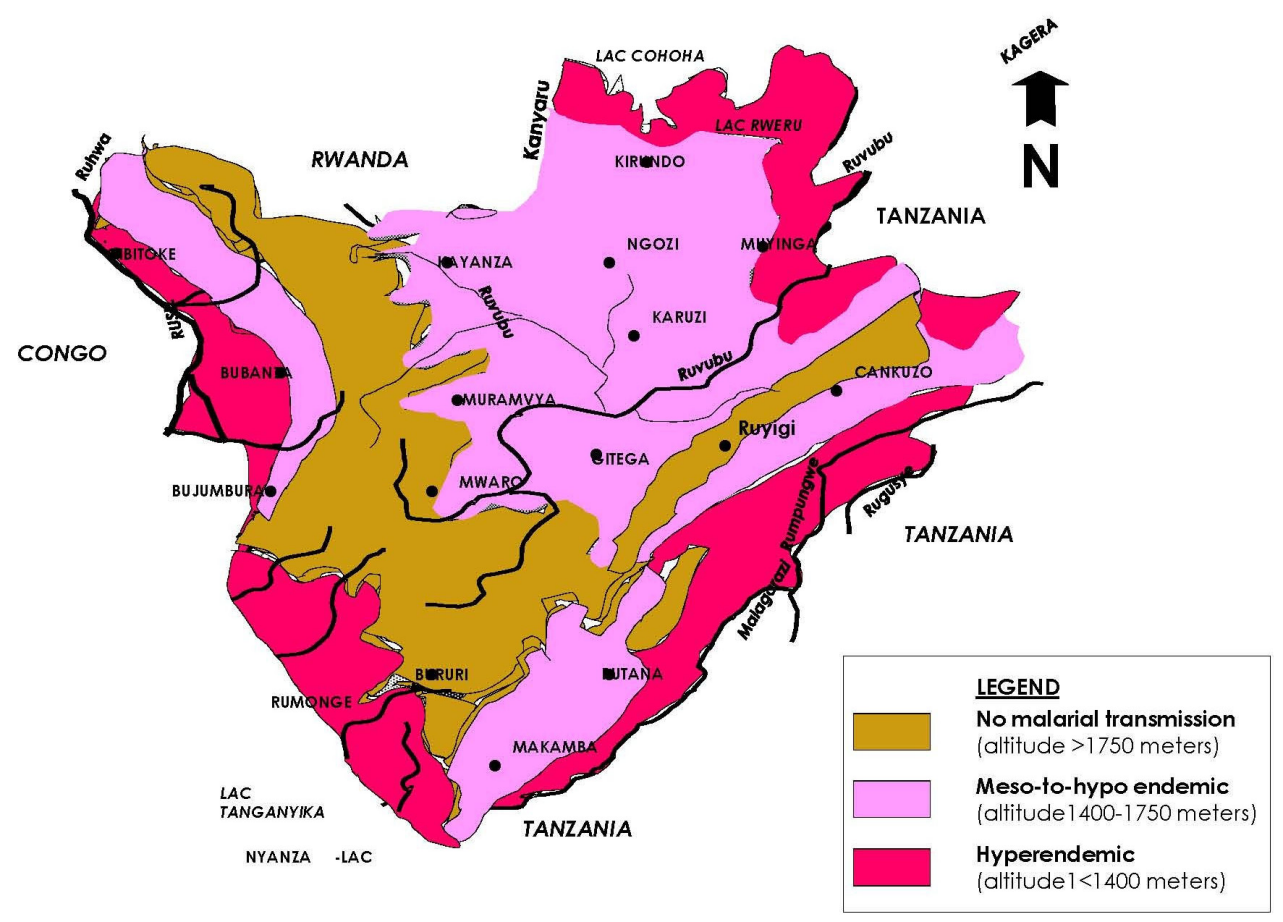
**Malaria transmission zones in Burundi**. *Source: NMCP Burundi epidemiological service*

A cross-sectional survey was conducted from February to March 2009, in three provinces of Burundi; Bujumbjura, Bururi, and Kayanza, representing different levels of risk for malaria transmission, hyperendemic (altitude <1,400 metres), meso-to-hypo endemic (altitude 1,400-1,750 metres) and areas of no malaria transmission (altitude >1,750 meters), respectively (Figure 1). In addition, these provinces were selected to provide an urban (Bujumbura) and rural (Bururi and Kayanza) population distribution. The majority of Burundians live in rural areas with only 9% living in urban areas [10]

### Selection of outlets

For each region, a list of public medicine outlets was compiled using a database obtained from the Ministry of Health in Burundi. A two-stage sampling approach was used to select the medicine outlets. First, from each of the three selected survey provinces, the major urban commune with the main public hospital was selected. Secondly, seven randomly selected public medicine outlets that were reachable within a day's travel from the main urban commune were selected. A matching number of private and NGO sector medicine outlets were selected by identifying one medicine outlet in each sector that was geographically closest to each public medicine outlet when possible. Private retail outlets were oversampled in Bujumbura and Bururi since there were few NGO medicine outlets (four and three, respectively) to match the number of public outlets. In Kayanza, all private (n = 6) and NGO (n = 3) outlets that existed in the region were sampled.

### Survey methodology

Adapted WHO and Health Action International (HAI) standardized methodologies for surveying medicine prices, availability, and affordability were used for this survey [11]. Pharmacists or medicine dispensers were recruited to participate in the survey and verbal consent was obtained from those who agreed to participate. For each anti-malarial found in the medicine outlet on the day of survey, trained data collectors recorded information on the brand name (originator and generic), price, manufacturer, and manufacturing company were recorded, as well as information on the medicine package type (loose pills, single pack, or hospital package), the availability of the anti-malarial as over-the-counter or prescription, and the number of anti-malarials sold in the last month. In addition, data on the medicine retailer's knowledge regarding WHO regulations and national anti-malarial treatment guidelines were collected. The price of the anti-malarial was originally collected in Burundian francs (FBu), and then converted to US dollars (US$). At the time of survey, US$1 was equivalent to 1,240 FBu.

### Data analysis

Data from the different sector medicine outlets were analysed separately and anti-malarials were stratified by treatment with first-line ACT (AS-AQ according to Burundian national policy and other WHO-recommended ACT), second-line quinine, or other non-policy anti-malarials. For the availability analysis, different dosage strengths of the same anti-malarial were combined to calculate the overall availability of that anti-malarial on the day of survey. Median prices of anti-malarials were computed if there were ≥4 medicines (following WHO-HAI methodology) identified for each anti-malarial category regardless of dosage or form; otherwise, the mean was calculated. Anti-malarials offered for free were not included in the analysis. For comparison, the median price ratios (MPR) of the anti-malarial median price to the current charge for a single treatment course of AS-AQ in public health facilities were calculated, except for quinine. The charge for a treatment course of AS-AQ dispensed in public sector facilities is US$0.16 (200 FBu). Since there is no government subsidized price for quinine, the MPR of public to private and public to NGO sectors were calculated.

Affordability was estimated using median anti-malarial prices and the average per capita annual income (US$140/per year or US$0.38/day) in Burundi and calculating the number of days' wages required to purchase a course of the anti-malarial [12]. Recently the President of Burundi declared that malaria treatment would be free-of-charge for children under five years of age and pregnant women, however, this policy was not in effect during the period of the survey [13].

## Results

In this study, a total of 70 medicine outlets, of which 24 (34.4%) were public sector hospitals, 36 (51.4%) private medicine outlets, and 10 (14.3%) NGO medicine outlets were surveyed in the regions of Bujumbura (n = 29), Bururi (n = 24), and Kayzanza (n = 17), respectively.

### Availability

The survey found anti-malarials stocked at all outlets. Nearly 90% of public and NGO outlets surveyed stocked AS-AQ, the recommended first-line treatment for uncomplicated malaria. However, only one-third of private outlets had AS-AQ stocked at the time of survey (Table 1). In all outlet types, the most common anti-malarial found was quinine and the tablet formulation was more prevalent than the injections. Other WHO recommended first-line ACTs (artemether-lumefantrine and dihydroartemisinin-piperaquine) were found in the private sector although they were not part of the Burundian anti-malarial policy. Chloroquine and sulphadoxine-pyrimethamine were not found in any outlets.

**Table 1:** Availability of anti-malarials on day of the survey by retail sector and geographical region

	Retail sector						Geographical region					
	Public (n = 24)	%	Private (n = 36)	%	NGO (n = 10)	%	Bujumbura (n = 29)	%	Bururi (n = 24)	%	Kayanza (n = 17)	%
ACT	21	87.5	12	33.3	9	90.0	15	51.7	11	45.8	16	94.1
AS-AQ^a^	21	87.5	12	33.3	9	90.0	15	51.7	11	45.8	16	94.1
Other first-line ACT^b^	-	-	2	5.6	-	-	-	-	2	8.3	-	-
**Quinine**	**23**	**95.8**	**28**	**77.8**	**9**	**90.0**	**27**	**93.1**	**22**	**91.7**	**16**	**94.1**
Quinine injection	17	70.8	23	63.9	9	90.0	21	72.4	16	66.7	12	70.6
Quinine tablets	23	95.8	28	77.8	9	90.0	25	86.2	19	79.2	16	94.1
Both quinine injection and quinine tablets	17	70.8	18	50.0	9	90.0	19	65.5	13	54.2	12	70.6
**Non-policy anti-malarials**	**1**	**4.2**	**14**	**38.9**	-	-	**9**	**31.0**	**3**	**12.5**	**3**	**17.6**
Amodiaquine	-	-	1	2.8	-	-	0	0.0	1	4.2	0	0.0
Dihydroartemisinin	-	-	3	8.3	-	-	2	6.9	1	4.2	0	0.0
Halofantrine	1	4.2	10	27.8	-	-	7	24.1	2	8.3	2	11.8
**Overall anti-malarial availability**	**23**	**95.8**	**28**	**77.8**	**9**	**90.0**	**27**	**93.1**	**22**	**91.7**	**16**	**94.1**
AS-AQ only	1	4.2	0	0.0	1	10.0	0	0.0	1	4.2	1	5.9
Quinine only	2	8.3	13	36.1	1	10.0	7	24.1	9	37.5	0	0.0
Non-policy antimalarials only	0	0.0	1	2.8	0	0.0	1	3.4	0	0.0	0	0.0
AS-AQ+quinine	20	83.3	8	22.2	8	80.0	13	44.8	10	41.7	13	76.5
AS-AQ+non-policy antimalarials	0	0.0	1	2.8	0	0.0	1	3.4	0	0.0	0	0.0
Quinine+non-policy antimalarials	1	4.2	9	25.0	0	0.0	6	20.7	3	12.5	1	5.9
AS-AQ+quinine+non-policy anti-malarials	0	0.0	3	8.3	0	0.0	1	3.4	2	8.3	0	0.0

ACT, artemisinin combination therapy; AS-AQ artesunate-amodiaquine (loose combination)

^a ^First-line treatment for uncomplicated malaria under Burundi's national antimalarial policy

^b ^Other first-line ACT antimalarials recommended by the WHO were identified (artemether-lumefantrine and dihydroartemisinin-piperquine)

Non-policy anti-malarials (amodiaquine, dihydroartemisinin, and halofantrine) were mainly found in the private sector (93.3%) and none in the NGO sector. More than 80% of public and NGO sector medicine outlets stocked both first-line and second-line policy recommended anti-malarials. In contrast, only one-third of private medicine outlets carried both first- and second-line treatment anti-malarials. Among the regions surveyed, availability of both these policy recommended anti-malarials was highest in the least malaria endemic area of Kayanza (Table 1). AS-AQ was available in about half of the outlets and quinine was available in over 90% of outlets in both Bujumbura and Bururi. The presence of non-policy anti-malarials was highest in the most endemic region of Bujumbura.

### Medicine prices

Overall, median prices of a treatment course of AS-AQ tablets, quinine injections, quinine tablets, and other non-policy anti-malarials were US$0.16, US$0.32, US$1.61, and US$8.55, respectively. Median prices of AS-AQ and quinine were lowest in the public sector and highest in the private sector (Table 2). The median price of ACT in the NGO sector was the same as the government subsidized price in the public sector. Quinine and non-policy anti-malarials were priced higher than AS-AQ. Although not part of national policy at the time of the survey, three public medicine outlets were dispensing free anti-malarials: one was in a prison and two public sector outlets had received donations.

**Table 2:** Median prices of anti-malarials being sold in the public, private and NGO sector

	Public sector		Private sector		NGO sector	
	**No. of anti-malarials**	**Median price (range) US$**	**No. of anti-malarials**	**Median price (range) US$**	**No. of anti-malarials**	**Median price (range) US$**

**ASAQ**	28	0.16 (0.16-0.16)	19	0.56 (0.16-2.82)	15	0.16 (0.08-2.42)
**Quinine**	53	0.59 (0.03-1.77)	73	1.09 (0.24-3.39)	25	1.19 (0.16-2.58)
Quinine injection	19	0.24 (0.12-0.48)	25	0.36 (0.24-1.61)	9	0.32 (0.16-0.81)
Quinine tablet	34	1.53 (0.03-1.77)	48	1.61 (0.32-3.39)	16	1.61 (0.51-2.58)
**Non-policy anti-malarials**	2	8.90 (0.32-11.29)^**a**^	19	8.55 (8.35-9.44)	0	

ACT, artemisinin combination therapies; US$, United States Dollars

Anti-malarials that were offered for free (n = 4; 1 ACT, 1 quinine injection, and 2 quinine tablets) were excluded from median price calculations.

^a^Mean was calculated since there were only two other anti-malarials available in the public sector. According to WHO-HAI methods, there should be at ≥4 medicines to calculate the median.

The highest MPR was non-policy recommended anti-malarials which was more than 50-fold in both the public and private sectors (Table 3). The private sector had the highest MPR for policy and non-policy anti-malarials compared to public and NGO sectors.

**Table 3:** Median price ratio of anti-malarials in public, private, and NGO sectors

	Public sector	Private sector	NGO sector
**ASAQ**^**a**^	1.0	3.5	1.0
**Quinine**	Ref^b^	2.1	2.0
Quinine injection	Ref^b^	1.5	1.3
Quinine tablets	Ref^b^	1.1	1.1
**Non-policy anti-malarials**^**a**^	55.6	53.4	-

ACT, artemisinin combination therapies

^a ^Median price ratio calculated based on government subsidized price of AS-AQ (US$ 0.16)

^b ^Median price ratios were calculated using the public sector price as reference for quinine since there is no government subsidized price: US$ 0.59 for quinine, US$ 0.24 for quinine injection, US$ 1.53 for quinine tablets.

**Figure 2:**
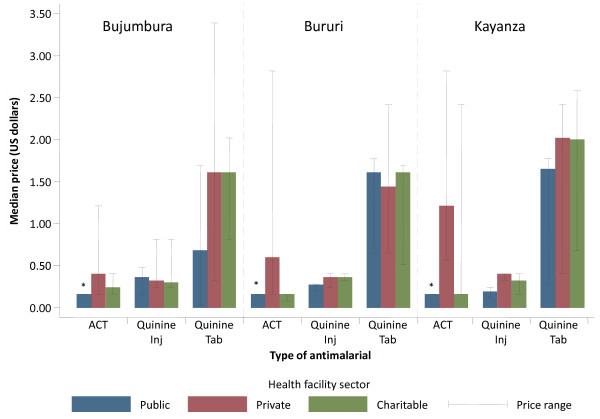
**Distribution of median prices of ACT and quinine being sold in the public, private and NGO sectors of Bujumbura, Bururi, and Kayanza**.

Figure 2 shows the median prices of anti-malarials found in the different medicine outlet sectors stratified by survey region. In general, private retail outlets in all three regions charged the highest prices for anti-malarials compared to public and NGO outlet types. AS-AQ was offered at the government subsidized price of US$0.16 in all public outlets surveyed across the three survey regions. The cost of AS-AQ in the private outlets was two-fold higher than the government subsidized price in Bujumbura and 7.5-fold higher in Kayanza. The cost of quinine injections was relatively similar in all three regions but the median price for quinine tablets was highest in Kayanza.

### Affordability

AS-AQ purchased within the public and NGO sectors were most affordable, representing 0.4 of a day's wage (Table 4). Quinine tablets cost four days worth of wages in all three sectors. The tablet formulation of quinine was less affordable compared to quinine injections. Non-policy recommended anti-malarials were least affordable, costing about one month's worth of wages.

**Table 4:** Number of days-wages to afford antimalarials in the public, private, and NGO sectors

	Public sector	Private sector	NGO sector
**AS-AQ**	0.4	1.5	0.4
**Quinine**	1.4	2.9	3.1
Quinine injection	0.6	1.0	0.8
Quinine tablets	4.0	4.2	4.2
**Non-policy antimalarials**	23.4	22.5	-

AS-AQ, artesunate-amodiaquine

## Discussion

Anti-malarials were accessible in all outlet types surveyed in Burundi. The results of this study showed high uptake of malaria treatment policy in post-conflict Burundi where nearly 90% of public and NGO outlets stocked AS-AQ, the recommended first-line treatment for uncomplicated malaria. Compared to other post-conflict countries, availability of anti-malarials in public facilities were 73.3% in Cambodia [14] and 84.2% in the Democratic Republic of Congo (DRC) [15]. Of the public outlets that stocked anti-malarials 88%, 24%, and 97% stocked first-line treatment ACT, respectively. The relatively high implementation of policy-recommended ACT may be a result of the government subsidy on AS-AQ; the relatively small size of the country to support its capacity to raise awareness of prevention and control activities and promote access to appropriate diagnosis and treatment; and the lack of local pharmaceutical manufacturers which may have limited the number of anti-malarials in the market. The results from this study also confirm other reports on the successful ban on the use of chloroquine and sulphadoxine-pyrimethamine as first-line treatment for uncomplicated *P. falciparum *malaria in Burundi [3].

Although AS-AQ was highly available, quinine was the most common anti-malarial in all retail sectors. Specifically, the private sector was four times more likely to carry quinine only than the public sector with no alternative anti-malarial available. This is of concern when 50% of the country's 126 private pharmacies are located in Bujumbura alone where malaria is hyperendemic [16]

A number of reasons could account for the dominant stock of quinine in the private sector. First, private sector medicine sellers view selling AS-AQ as unprofitable, since it is highly subsidized in the public sector and they are not able to match those prices. Consequently, they may prefer to stock quinine which they can retail at a competitive price. Secondly, quinine has been available in Burundi since the 1940s and is one of the few anti-malarials manufactured in-country, making it more popular as an anti-malarial and more easily accessible than AS-AQ, which is imported. Thirdly, despite WHO recommendations for other ACT to be adopted as second-line, the policy in Burundi as well as in 29 other African countries continues to be oral quinine monotherapy as second-line therapy [17], which could possibly be negatively affecting uptake of AS-AQ as first-line. Finally, private medicine sellers may not have received adequate training for malaria treatment and management, as 96% of public and 90% of NGO sector medicine sellers in this study were familiar with the indication of ACT use as being for uncomplicated malaria, compared to 39% in the private sector.

Although familiarity was lower for indication for use of quinine in cases of complicated malaria across all three sectors, it was the lowest in the private sector (58.3% in the public, 40% in the NGO, and 25% in the private, respectively). Since the mission hospitals in the NGO sector facilities receive government support, they honour the same policy for anti-malarials and have in effect received adequate training and incentives to dispense ACT, whereas the private sector does not have this incentive. Similar to the NGO sector, the private sector needs to engage in the ACT implementation process and be provided with adequate training to ensure correct dispensing of anti-malarials as well as sufficiently stocking ACT in their facilities.

Alarmingly, several non-policy anti-malarials (amodiaquine, dihydroartemisinin, and halofantrine) were identified, which were present almost exclusively in the private sector. Specifically, halofantrine was available in over one-quarter of private sector outlets without prescription, which is a cause for concern given the association of adverse cardiovascular effects with this drug [18] and the potential for cross-resistance with lumefantrine (LUM) and mefloquine (MQ) [19]. This could lead to devastating effects on the possibility of future distribution of ACT containing lumefantrine or mefloquine in Burundi. The presence of these non-policy anti-malarials requires more effective measures to counter price gouging, smuggling, and counterfeiting if widespread distribution of new fixed-dose AS-AQ is to be achieved [20].

Besides identifying other non-policy anti-malarials being highly prevalent in the private sector, they were also highly prevalent in the region of Bujumbura, the most endemic malaria transmission zone in Burundi. In the high altitude region of Kayanza, the high availability of AS-AQ compared to non-policy anti-malarials may reflect the disincentives in distributing drugs to these hard-to-reach communities other than government regulated anti-malarials. In addition, since Bujumbura is the capital city, the high concentration of private outlets carrying non-policy anti-malarials is not surprising since accessibility to imports is much easier in this urban area compared to the other two rural regions in this study. Also, retailers may be more likely to inflate prices since persons of higher socioeconomic status tend to live in urban areas [6,21] and are more likely to be able to afford them.

Since the non-profit NGO sector is supplied with AS-AQ by government and adheres to the subsidy policy, it is not surprising that the pricing of ACT was found to be similar in the public and NGO sectors. As compared to the DRC, AS-AQ in Burundi is affordable (US$3.20 *vs. *US$0.16) [15], owing to the government's effort to subsidize the ACT price. However, the price differential between the NGO and public sectors for second-line treatment medicines was similar to that between the private and public sector, highlighting the need to consider the same affordable pricing regime for not only first-line treatment but also second-line treatment. Given the 3.5-fold price differential of AS-AQ in the private sector compared to the public sector, there is a need to make the pricing of ACT in the private sector comparable to that in the public sector. Innovative financing mechanisms should be considered to incentivize the private sector towards retailing of ACT, such as what has been done in neighbouring Rwanda [22].

Although AS-AQ appears to be affordable in the public sector, it still costs nearly half a day's wages in Burundi, and in the private sector, the cost is 1.5 days worth of wages. Although substantial progress has been made to make AS-AQ available in the public health system of Burundi, it is clear that those who seek treatment in the private sector are less likely to access ACT, but they will continue to purchase non-policy recommended and potentially harmful or ineffective anti-malarials, or use the cheaper quinine monotherapy. This is of concern, as it is known that most patients seek treatment in the private sector rather than the public sector given the long distance to travel to, long waiting times at, and poor availability of drugs in the public sector [23]. Further study is needed to evaluate the role of the private sector in providing treatment for patients in Burundi, so that an effective evidence-based intervention could be considered to effectively increase AS-AQ accessibility.

There are several limitations in this study. Firstly, although the study objective was not to evaluate the factors determining the price of anti-malarials, the study lacked comparator drugs (i.e. other essential medicines) with the anti-malarials evaluated, which made it difficult to understand whether price variations found reflect the general prices of all drugs distributed in the retail outlet or the findings were specific to the class of anti-malarials. For example, if the price mark-ups of AS-AQ were significantly higher than other essential medicines, it would be important to understand and address the factors that influence these retail price gaps. Secondly, although we evaluated the availability of AS-AQ and other anti-malarials, it remained unclear which specific anti-malarial was dispensed to patients as some private medicine outlets stocked up to 10 brands of medicines. It would be relevant to conduct a "mystery shopper" survey in order to evaluate any potential bias that might have arisen from directly questioning the medicine dispensers on their knowledge and practice. Thirdly, the availability of anti-malarials should be interpreted with caution since it is unclear how the selection of outlets within one day's travel from the main urban public outlet in the region and the disproportionate distribution of medicine outlets sampled in the private and NGO sectors might have under- or over-estimated the accessibility of AS-AQ and other anti-malarials. It is possible that in hard-to-reach rural areas, ACT may not offer a profitable market for the private sector since it may require a more complicated distribution chain from the supplier to the retail outlet [21].

## Conclusions

Preliminary results of this study prompted a meeting with a panel of experts to convene in September 2009, and proceedings from this meeting have been published [24]. The high availability of national policy recommended AS-AQ in the public and NGO sector outlets support the capacity of these two sectors to adopt policy into practice and gives high promise in the successful implementation of fixed-dose ASAQ in Burundi. However, the results also confirm studies in Africa, where there is an ever-increasing role of the private sector market in having a large impact on ACT policy implementation [25-27]. The MoH of Burundi is taking steps to improve accessibility of AS-AQ by considering similar pricing for fixed-dose ASAQ in both the public and private sector. However, the challenge remains in engaging with the private sector to make AS-AQ and other policy recommended anti-malarials widely available in their outlets and at an affordable price. Innovative strategies like the Global Fund's Affordable Medicines Facility-Malaria (AMFm) initiative are being piloted and evaluated and are aimed at subsidising high quality anti-malarials at the manufacturer level in both the public and private sectors [28,29]. If successful, there is the potential to increase widespread access to affordable ACT, bridge the gap between the public and private sectors, and reduce the use of inappropriate treatments.

## Competing interests

The authors declare that they have no competing interests.

## Authors' contributions

JHA conceived and designed the study; initiated, conducted and coordinated the study; and contributed to the drafting of the manuscript. GD conceived the study, and participated in its design and coordination; also, helped to draft the manuscript. SBN conducted the statistical analysis and contributed to the drafting of the manuscript. IB participated in the design of the study and helped conduct the statistical analysis; also, contributed to the drafting of the manuscript. PEK reviewed and discussed the study results; conducted the dissemination of results at country level; and contributed to the drafting of the manuscript. BD helped in designing the study; reviewed and discussed the study results and helped conduct the dissemination of results at country level; also contributed to the writing of the manuscript. JK helped in designing, conducting and coordinating the study; reviewed and discussed the study results and helped conduct the dissemination of results at country level; also contributed to the drafting of the manuscript. LN reviewed and discussed the study results and helped conduct the dissemination of results at country level; also contributed to the drafting of the manuscript. KSL performed the analysis of the data and drafted the manuscript. JRK made contributions to the study conception and design; also contributed to the drafting of the manuscript. All authors have read and approved the final manuscript.
